# Supramolecular Control of Fullerene Recognition and Reactivity through Nanocapsule Confinement

**DOI:** 10.1021/acs.accounts.6c00055

**Published:** 2026-04-01

**Authors:** Valentina Iannace, Xavi Ribas

**Affiliations:** Institut de Química Computacional i Catàlisi (IQCC) and Departament de Química, Universitat de Girona, Girona 17003 Catalonia, Spain

## Abstract

Fullerenes have a wide range of applications across biomedicine, electronics, and nanotechnology, yet their broader application depends on the ability to control their molecular recognition and chemical reactivity. Supramolecular host–guest chemistry offers powerful opportunities in this context by enabling selective encapsulation and modulation of reactivity within confined environments. Since their discovery, substantial efforts have been devoted to developing efficient purification protocols to obtain high-purity fullerenes (particularly higher fullerenes C_n_, *n* > 70) from fullerene soot, thereby avoiding tedious and costly chromatographic separations. The use of molecular receptors for fullerene purification via host–guest interactions affords good selectivity, requires no specialized equipment, and enables recyclable systems through careful host design. To date, most supramolecular receptors have demonstrated differential recognition of fullerenes through distinct binding affinities, while translation of such selectivity into practical purification and, more importantly, into predictable control over reactivity remains a central challenge. Indeed, to further advance fullerene chemistry, access to isomer-pure polyfunctionalized fullerenes is essential. However, conventional functionalization methods typically yield mixtures of multiple adducts with poor regioselectivity, and chromatographic purification alone is often insufficient. An alternative strategy involves confining fullerenes within host cages that act as supramolecular masks, selectively shielding part of the fullerene surface, which has emerged as an effective approach for the direct synthesis of isomer-pure polyadducts.

This Account describes our development of tetragonal prismatic nanocapsules as a unified platform for the control of fullerene recognition and reactivity. Through careful host design and detailed investigation of binding sites and binding modes, these nanocapsules enable the selective encapsulation of fullerenes with different shapes and sizes (C_60_, C_70_, C_84_, and fullertubes) while also providing a confined environment that governs subsequent chemical transformations, such as the regioselective functionalization of C_60_ and C_70_.

In this context, supramolecular confinement not only enables selective fullerene binding but also modifies the guest’s accessible surface area, thereby enabling regioselective functionalization via a supramolecular mask strategy and introducing a new level of selectivity. By systematically correlating nanocapsule structure with binding behavior and reaction outcomes, we demonstrate how cavity size matching, window accessibility, and postbinding host–guest interactions can be leveraged to achieve selective encapsulation and predictable regioselectivity in fullerene functionalization. These studies show the potential of the supramolecular control over selectivity and reactivity in highly symmetric guests.

The principal remaining challenges include achieving high selectivity for specific substrates and effective discrimination among multiple competing guests through distinct, well-defined binding modes, as well as extending confinement-controlled reactivity to increasingly complex substrates. These challenges will be addressed by developing novel supramolecular containers capable of predictable recognition, enhanced selectivity, and precise control of reactivity within confined environments.

## Graphical abstract

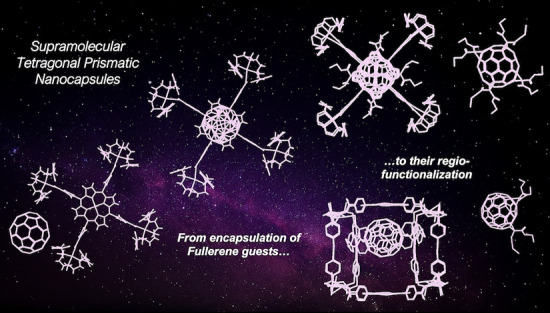

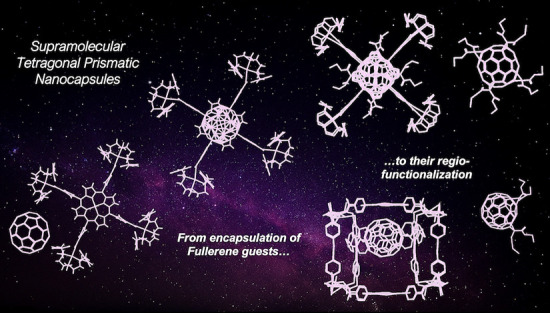

## Key References

-

García-Simón, C. ; et al. Sponge-like molecular cage for purification of fullerenes. Nat. Commun. 2014, 5, 5557. Our first supramolecular tetragonal prismatic nanocapsule capable of encapsulating C_60_ and C_70_ was reported.25424201 10.1038/ncomms6557
-

Fuertes-Espinosa, C. ; et al. Supramolecular Fullerene Sponges as Catalytic Masks for Regioselective Functionalization of C_60_
. Chem 2020, 6, 169. The potential of the Supramolecular Mask Strategy for the obtention of regioselective Bingel reactions inside the nanocapsule was demonstrated.
-

Ubasart, E. ; et al. A three-shell supramolecular complex enables the symmetry-mismatched chemo- and regioselective bis-functionalization of C_60_
. Nat. Chem. 2021, 13, 420. Unprecedented control of fullerene reactivity through multilevel supramolecular confinement in a hierarchical three-shell supramolecular complex was demonstrated.33859394 10.1038/s41557-021-00658-6
-

Iannace, V. ; et al. Regioswitchable Bingel Bis-Functionalization of Fullerene C_70_ via Supramolecular Masks. J. Am. Chem. Soc. 2024, 146, 5186. Mask strategy for the Bingel regio-functionalization was successfully applied to nonspherical C_70_ fullerene38311922 10.1021/jacs.3c10808PMC10910506.

## 1 Introduction

Fullerenes are an allotropic form of carbon consisting of discrete molecules with a defined number of C_sp2_ atoms in a highly symmetric structure. First observed by Kroto and Smalley in 1985,^1^ their discovery enabled multiple applications in materials science, electroactive materials in solar cells,^2^ superconducting materials,^3^ and biomedical applications.^4^ Since their discovery, efforts have focused on understanding and controlling their molecular recognition and chemical reactivity, particularly isolating and functionalizing specific fullerene structures within complex mixtures and controlling the regio- and iteroselectivity of their chemical modification. Achieving such control remains a central challenge, owing to the similar sizes and shapes of different fullerenes and to the high symmetry and uniform reactivity of their carbon frameworks. Crude products from the arc-discharge method^5^ contain mixtures of fullerenes and other carbon allotropes. Purification usually relies on chromatographic techniques, which are often expensive and time-consuming, hampering the isolation of specific fullerenes with high purity, especially higher ones (C_*n*_, *n* > 70).^6^ On the other hand, functionalization of bare fullerenes typically yields intractable mixtures of multiadducts with poor regioselectivity, limiting the development of fullerene chemistry, as functionalization is essential for tuning the chemical and physical properties of fullerenes, and access to pure polyfunctionalized fullerenes is highly desirable for materials applications.^7^ Consequently, the development of improved purification methodologies is both challenging and highly desirable. Supramolecular receptors for fullerene encapsulation with differential recognition (through variations in binding affinity and size complementarity) offer an attractive alternative,^8^ providing high selectivity, recyclability, and no need for specialized equipment. Encapsulation in supramolecular hosts also enables solubilization and chemical modification of fullerenes, even in otherwise inaccessible polar solvents.

Motivated by these challenges, our group has developed a family of porphyrin-based tetragonal prismatic supramolecular nanocapsules,^9^ which serve as versatile platforms for the control of fullerene recognition and reactivity. Encapsulation within these nanocapsules not only allows selective recognition of fullerene guests of different shapes and sizes, but also enables control over regio^10^- and itero^11^-functionalization of the encapsulated fullerenes. Strikingly, our supramolecular mask strategy provides an effective means to control regioselectivity while preventing overfunctionalization. This approach relies on the careful design of a supramolecular host with high affinity for fullerenes, followed by functionalization of the resulting host–guest complex. In this confined environment, chemical modification occurs exclusively at the exposed surface of the fullerene that remains accessible beyond the supramolecular mask.

Over the past five years, we have applied this strategy to multiple functionalization reactions, including Bingel^12^ and Diels–Alder^13^ reactions, and to different fullerene guests, such as C_60_,^14^ and C_70_.^15^ Furthermore, this approach can be extended to more sophisticated three-shell supramolecular assemblies.^16,17^ In this context, supramolecular encapsulation and masking represent complementary aspects of a unified strategy: the use of nanocapsule confinement to achieve predictive control over selectivity and reactivity in highly symmetric guests.

Supramolecular Mask Strategy (SMS) complements earlier approaches for controlling fullerene regioselectivity. Tether-directed remote functionalization approach^18^ represents one of the first “supramolecular-like” strategies using a rigid spacer covalently attached to two functionalization moieties to direct the functionalization pattern on the fullerene surface. While effective for both C_60_ and C_70_, it requires multistep tether synthesis and removal, when possible.^19^ On the other hand, some recent confined-based systems illustrate the diversity of supramolecular approaches to fullerene functionalization. Clever and co-workers^20^ reported a bowl-shaped host with a single aperture enabling selective monofunctionalization. Nitschke and co-workers,^21^ on the other hand, reported a self-assembled cage that acts simultaneously as a host and as a stereochemical template that transfers the stereochemical information to the product. Also, covalent cage architectures with multiple small windows (Beuerle and co-workers^22^) restrict access to defined regions of the fullerene surface and allow partially regioselective tris-Prato addition on encapsulated C_60_. Compared to these systems, our self-assembled supramolecular tetragonal prismatic nanocapsules function as nanoscale reaction environments integrating molecular recognition and confinement-controlled reactivity. Combined experimental studies and Molecular Dynamics simulations^23^ provided insight into the host–guest interactions and the capabilities of these nanocapsules as supramolecular masks. By correlating structural features of the host structure with binding behavior and reaction outcomes, we define general principles for the supramolecular control of selectivity and reactivity in confined nanocapsule systems. These nanocapsules combine adaptive encapsulation with tunable window geometry and the possibility of obtaining multilayer confinement. Together, these complementary strategies highlight the growing potential of supramolecular confinement as a general platform for controlling reactivity in highly symmetric guests.

## 2 Design, Self-Assembly of Tetragonal Prismatic Nanocapsules and Derived Library

Molecular receptors for fullerene selective encapsulation through host–guest interactions have attracted considerable attention over the past decades.^8^ Metallo-supramolecular receptors are particularly appealing, as metal–ligand coordination provides a versatile and highly controllable design of fullerene hosts.^24^

Achieving stable host–guest complexes requires design optimization to maximize the cumulative strength of typically weak noncovalent interactions through cooperative binding. Careful analysis of the host cavity is therefore essential for identifying and optimizing guest binding sites. In particular, several key factors must be considered: (a) structural complementarity, including size and shape matching; (b) electronic complementarity, with extended π-systems being especially advantageous, as they can act as π-donors toward the π-accepting fullerenes.

To satisfy the second requirement, we employed metallo-porphyrin units, relying on their extended π-conjugated surfaces and their strong interactions with fullerenes.^25^ Accordingly, two-faced Zn^2+^ porphyrins separated by a well-defined distance were incorporated into a rigid 3D architecture (Figure 1). Size complementarity is governed by the separation between the porphyrins, tunable by varying the synthons that connect them (Figure 1a). All nanocapsules are constructed via metal-directed self-assembly of two tetracarboxylated Zn^2+^ porphyrins and four hexaaza macrocyclic bimetallic M^2+^ complexes (M = Pd, Cu).^26^ The resulting assemblies are formed through η^1^-O monodentate coordination between the carboxylate groups of the porphyrins and the M^2+^ centers (Figure 1b). In the final structure, the two Zn^2+^ porphyrins act as the caps of the nanocapsule and are arranged in a parallel fashion, while the four macrocyclic complexes act as walls, with their length determining the separation between porphyrins. The aromatic panels in the macrocyclic walls help stabilize guests through noncovalent interactions. The architecture adopts a *D*_4_ *h* tetragonal prismatic geometry, defined by the eight carboxyphenyl vertices. Notably, coordination at the eight M^2+^ vertices renders the nanocapsules octacationic, thereby imparting high solubility in polar solvents with selected counteranions.

**Figure 1:**
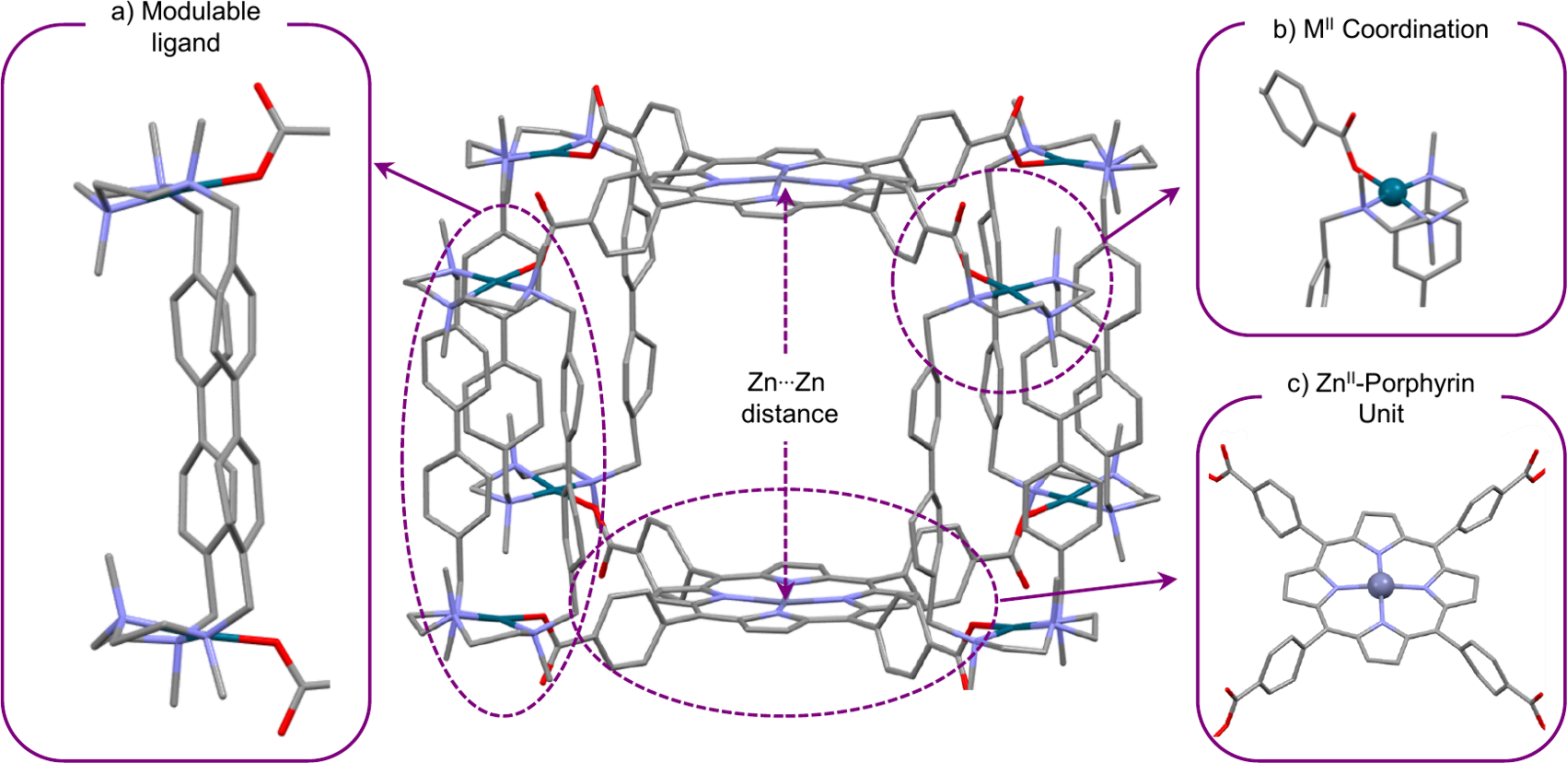
(a–c) Key features of our designed supramolecular tetragonal prismatic nanocapsules.

This class of tetragonal prismatic nanocapsules allows structural tuning through modification of the synthons. The nature of the M^2+^ center can be varied to modulate the flexibility of the coordination bonds, thereby influencing encapsulation and release dynamics. We have primarily explored Pd^2+^- and Cu^2+^-based systems, with the latter yielding more flexible nanocapsules. Counterion exchange can be readily achieved and has a pronounced impact on host solubility and overall chemical behavior. In most of our work, the counterion of choice is BArF^–^ (tetrakis­[3,5-bis­(trifluoromethyl)­phenyl]­borate), which imparts excellent solubility in MeCN, DCM, and THF. Additionally, the length of the macrocyclic complexes can be systematically varied to tune the separation between the Zn^2+^ porphyrins, controlling the size of the internal cavity, while preserving its tetragonal prismatic geometry (Figure 2).

**Figure 2:**
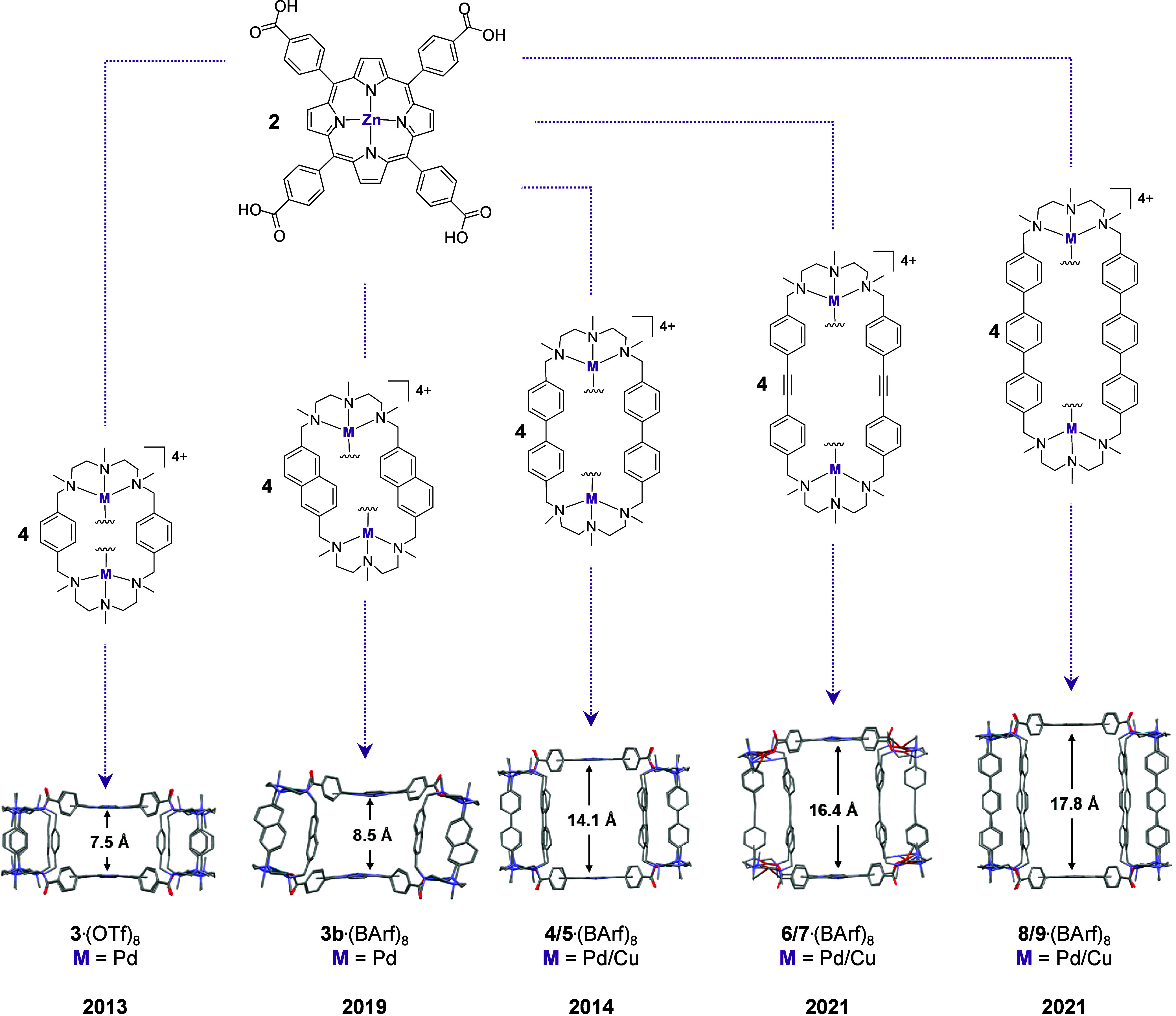
Our library of nanocapsules with progressively larger inner cavities, depending on the molecular clip employed, developed over recent years, along with their corresponding nomenclature (depicting either crystal structures or DFT-optimized structures).

## 3 Fullerene Encapsulation: Host-Guest Chemistry and Selectivity

Several hosts have been designed to encapsulate fullerenes through supramolecular interactions with specific recognition motifs.^8^ Our tetragonal prismatic nanocapsules were evaluated as efficient fullerenes receptors, and their exquisite selectivity toward fullerenes of different shapes and sizes was carefully examined.

### 3.1 Encapsulation of Fullerenes C_60_ and C_70_

In this section, nanocapsules **3b**·(BArF)_8_ ^27^ and **4**·(BArF)_8_ ^9^ are discussed as excellent hosts for fullerenes (Figure 2). The first nanocapsule we reported, **3**·(OTf)_8_,^26^ has a relatively small inner cavity (Zn···Zn distance = 7.5 Å), which is insufficient to accommodate fullerenes. In contrast, **3b**·(BArF)_**8**_ features a slightly larger inner cavity (Zn···Zn distance = 8.5 Å) thanks to its enlarged 2,6-disubstituted naphthalene-based macrocyclic ligand. By mixing equimolar solutions of **3b·**(BArF)_8_ in MeCN and C_60_ in toluene, the corresponding **C_60_⊂** **3b**·(BArF)_**8**_ host–guest complex was obtained, as confirmed by high-resolution mass spectrometry (HR-MS). UV–Vis titration data yielded an association constant of *K*_a_ = 1.29 (±0.42) × 10^5^ M^–1^. Remarkably, **3b**·(BArF)_8_ exhibited significantly lower affinity for C_70_ compared to C_60_, in contrast to most molecular receptors, which typically display higher binding for larger fullerenes (C_*n*_, *n* > 70) over C_60_. This size-selectivity can be rationalized by the Zn···Zn distance fluctuations of the empty nanocapsule—its so-called “breathing” ability (Figure 3c). Encapsulation of C_70_ induces severe distortion in **3b**·(BArF)_8_, forcing the Zn···Zn distance beyond the range allowed by the cage’s flexibility. In contrast, the Zn···Zn distance for encapsulated C_60_ remains within the permissible range, consistent with the nanocapsule’s structural adaptability.

In contrast, the **4**·(BArF)_8_ nanocapsule features a larger macrocyclic linker based on a biphenyl ligand, resulting in a Zn···Zn distance of 14.1 Å in the crystal structure. This structure exhibits no strong size-selectivity toward a specific fullerene but shows exceptional breathing ability and structural flexibility. Rapid inclusion of C_60_ was observed, with an association constant of *K*_a_ = 2.8 (±0.6) × 10^7^ M^–1^. The crystal structure of the host–guest complex revealed a shrunken Zn···Zn distance of 13.1 Å. Parallel experiments with C_70_ also showed fast inclusion, with an even higher *K*_a_ = 3.98 (±2.0) × 10^8^ M^–1^. XRD analysis indicated that the ellipsoidal C_70_ aligns its semimajor axis parallel to the porphyrin planes, and the Zn···Zn distance contracts to 13.7 Å. Moreover, leveraging the large cavity of **4**·(BArF)_8_, partial encapsulation of C_84_ was also achieved.^28^ The remarkable adaptability of **4**·(BArF)_8_ (Figure 3 c) toward guests of different sizes (C_60_, C_70_, C_84_) stems from the flexibility of the Pd–carboxylate coordination, which holds the supramolecular structure together while modulating the size of the inner cavity.

### 3.2 Dynamic Reconstruction of the Encapsulation Process

To gain insights into the fullerene recognition and binding mechanism, as well as the origin of the size-selectivity of **4**·(BArF)_8_, the encapsulation process was dynamically reconstructed using computational methods.^23^ Molecular Dynamics (MD) simulations of **4**·(BArF)_8_ revealed exceptional flexibility in solution, particularly in the twisting of the four macrocyclic clips that define the entrance gates. This flexibility translates into Zn···Zn distances ranging from 11.3 Å to 15.8 Å (average 13.6 ± 0.5 Å), closely matching the 14.1 Å observed in the XRD crystal structure. The intrinsic adaptability of the supramolecular capsule is key to explaining its affinity for fullerenes of different sizes. MD simulations of C_70_ encapsulation showed that the capsule undergoes compression to stabilize the fullerene inside, enhancing π–π interactions between C_70_ and the porphyrin moieties and further stabilizing the host–guest complex. The simulations also highlighted the critical role of the phenyl rings connecting the porphyrin units to the Pd-bound carboxylates: at each of the four entrances, four phenyl rings act as gates that can adopt open or closed conformations to allow fullerene entry (Figure 3a. Once C_70_ is fully enclosed, these phenyl rings stabilize it at the cavity center through C–H···π interactions, preventing displacement (Figure 3b). For C_60_, similar transient intermediates are observed; however, the nanocapsule undergoes greater compression upon binding due to the smaller size of C_60_ relative to C_70_. As a result, C_60_ exhibits higher mobility inside the cavity, indicative of weaker π–π interactions, and its encapsulation requires greater deformation of the nanocapsule (Figure 3c), which reduces the overall structural stability. Consequently, C_60_ unbinding is favored relative to C_70_, consistent with the observed selectivity.

### 3.3 Encapsulation of Higher Fullerenes and Fullertubes

Observing the partial inclusion of C_84_ in **4**·(BArF)_8_, we envisioned that modular tetragonal prismatic nanocapsules could be redesigned to expand encapsulation, providing a strategy for the purification of C_84_ and higher fullerenes.^29^ Since C_84_ exhibited poor affinity for **4**·(BArF)_8_, a larger nanocapsule based on a 1,2-diphenylethyne macrocycle (**6**·(BArF)_8_, Zn···Zn distance = 16.8 Å) was envisioned as a more suitable host (Figure 2). When **6**·(BArF)_8_ was exposed to 100 equiv of a fullerene extract (70% C_60_, 28% C_70_, and 2% higher fullerenes from C_76_ to C_96_, with C_84_ being the most abundant at 0.7%), selective encapsulation of C_84_ was observed after 7 days (detected by HRMS), indicating excellent size and shape complementarity. Upon release of C_84_ through nanocapsule disassembly, a 125-fold enrichment (from 0.7% to 86%) of C_84_ was achieved (Figure 4).^30^

We next sought to improve the selective binding of larger fullerenes by evaluating the analogous nanocapsules **8**·(BArF)_8_ (Pd-based) and **9**·(BArF)_8_ (Cu-based), both characterized by a large Zn···Zn distance of 17.8 Å. Unfortunately, encapsulation of higher fullerenes was unsuccessful, as the cavities were too large to effectively stabilize any of the higher fullerenes present in the extract. In contrast, the dumbbell-shaped azafullerene (C_59_N)_2_ was successfully encapsulated by these terphenyl-based nanocapsules, **8**·(BArF)_8_ and **9**·(BArF)_8_,^30^ demonstrating that precise host design is crucial for the selective encapsulation of fullerenes with different sizes and shapes.

So far, we focused on the size-selectivity of the nanocapsule toward fullerenes differing in diameter—namely C_60_ and C_70_ (d = 7 Å, short axis) and C_84_ (d = 8.5 Å)—which relies on the ability of the nanocapsule to contract or expand the Zn···Zn distance to adapt to guests of different sizes. We then asked what would occur upon encapsulation of a tubular fullerene-type species, that is, a guest in which the diameter remains constant while the length increases along an axis parallel to the porphyrin planes.

[5,5]­Fullertubes,^31^ a recently discovered family of fullerenes with a fixed diameter of 7 Å and varying nanotubular lengths (from 10.4 Å to 15.4 Å), provided an ideal probe to evaluate the influence of guest length on supramolecular encapsulation. Because the short-axis diameter of these tubular guests matches that of spheroidal C_60_ and ellipsoidal C_70_, **4**·(BArF)_8_ was selected as the most appropriate host. Encapsulation of C_90_ yielded an association constant of *K*_a_ = 1.5 × 10^5^ M^–1^, while C_100_ showed a higher value of *K*_a_ = 4.4 × 10^5^ M^–1^. As these two fullertubes differ only in length, the observed 2.6-fold increase in binding affinity for C_100_ can be directly attributed to its greater length. Competitive experiments using mixtures that also contained C_120_ ^32^ and C_130_ ^33^ (in traces) revealed a more favorable encapsulation of C_120_ over both C_90_ and C_100_, allowing us to infer an even higher association constant for C_120_. Strikingly, the detection of C_130_ among the released guests demonstrated that this longer species was also successfully encapsulated with even higher affinity, despite its trace abundance in the starting mixture. To complete the picture, molecular dynamics (MD) simulations were performed to investigate both the bound state and the host–guest dynamics. The shorter C_90_ remains centered within the cavity and undergoes free rotation due to its smaller size, resembling the behavior observed for C_70_, whereas clip-based *clamping* is observed for the longer fullertubes. By *clamping* (Figures 3c), we refer to the inward movement of the capsule clips that tightly retain the fullertube, reducing the window size and thereby enhancing host–guest interactions. For longer fullertubes, rotational motion within the cavity is suppressed due to their extended length. In the case of C_130_, a strong dual-window clamping effect maximizes host–guest contacts, resulting in particularly tight encapsulation of the elongated guest (Figure 4). Overall, this study highlights the crucial role of the capsule clips in the recognition of tubular guests,^34^ unveiling a new subtle tuning of the encapsulation capabilities in our nanocapsules.

**Figure 3:**
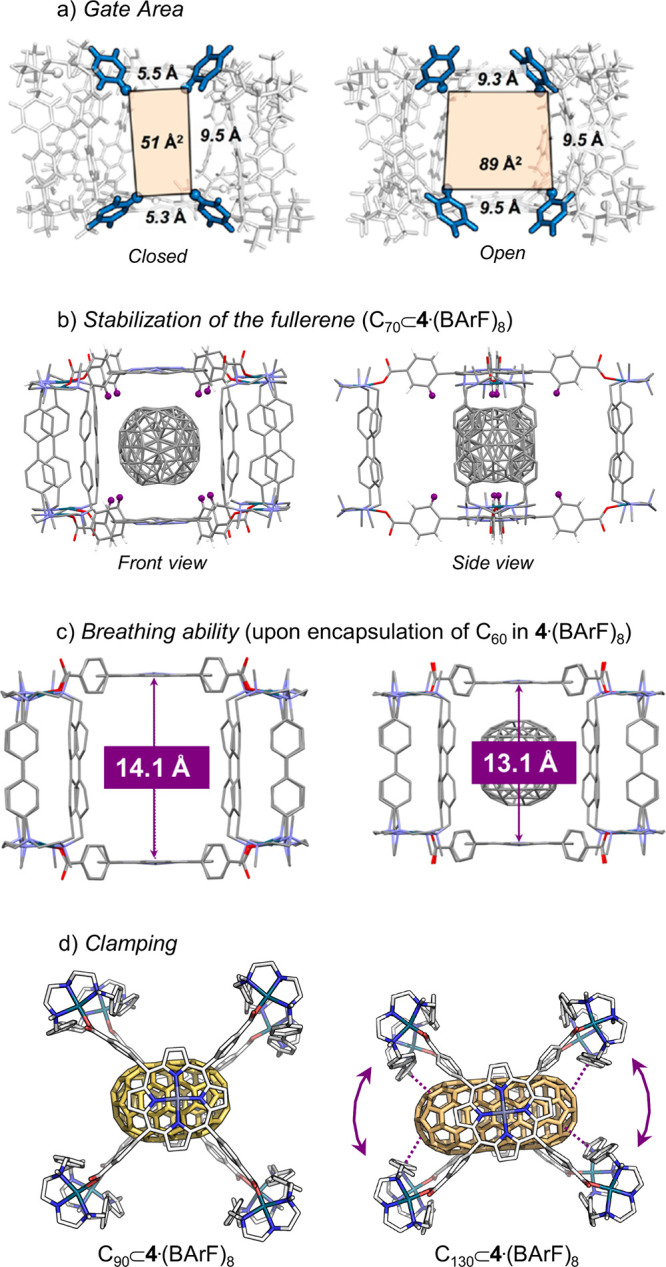
(a) Role of the porphyrin phenyl rings in opening and closing the nanocapsule gates. (b) Stabilization of the guest via C–H···π interactions (H atoms shown in purple). (c) Breathing ability of the nanocapsule: changes in Zn···Zn distances upon guest encapsulation. (d) Clamping of tubular guests (fullertubes): reduction of the entrance window size by clamping.

**Figure 4:**
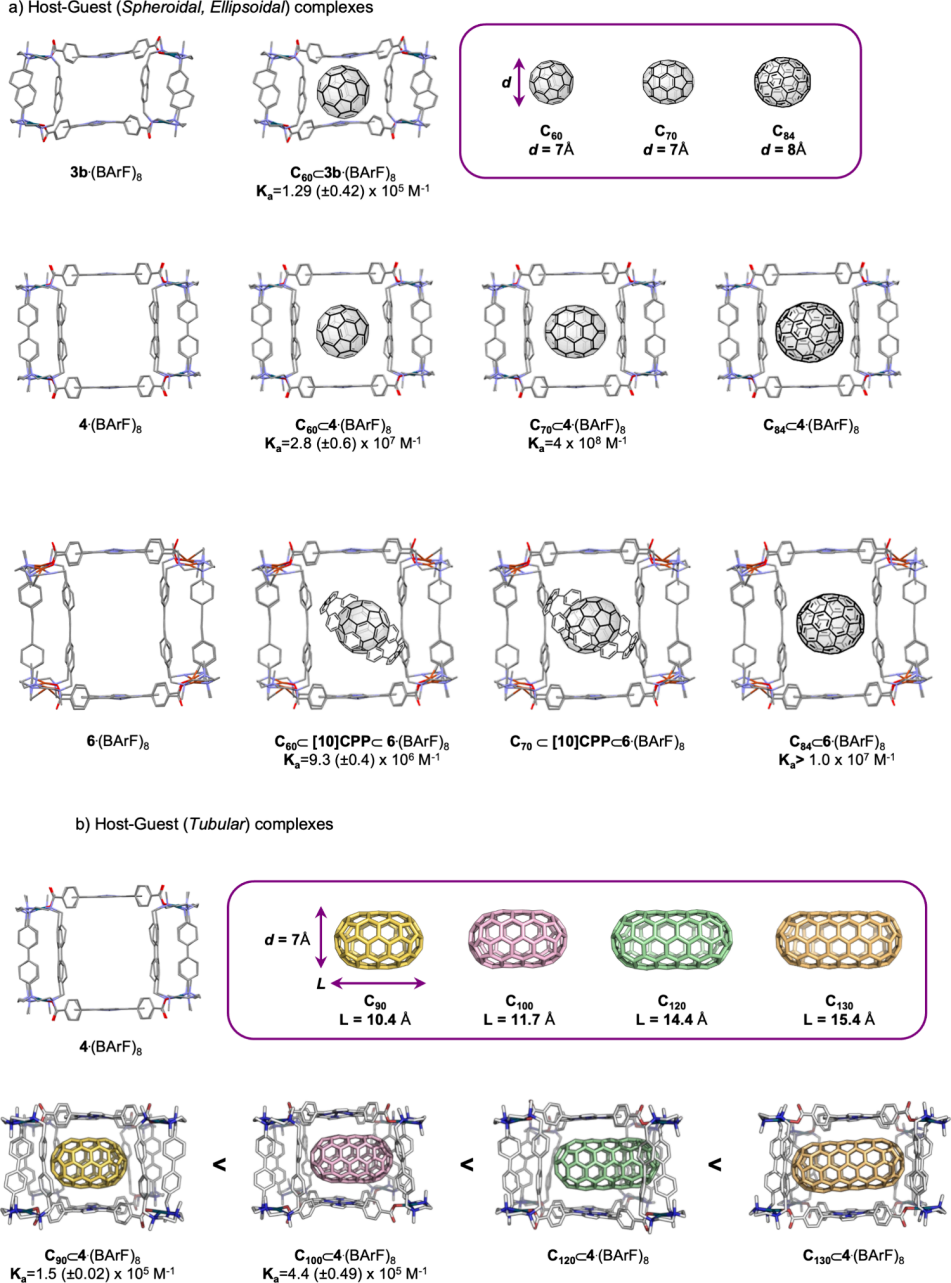
(a) Host–guest complexes formed with spheroidal and ellipsoidal fullerenes using nanocapsules **3b**·(BArF)_8_, **4**·(BArF)_8_, and **6**·(BArF)_8_, along with their corresponding association constants (*K*_a_). (b) Trend in *K*_a_ values for the encapsulation of tubular guests (fullertubes) as a function of guest length.

## 4 Fullerene Functionalization: Supramolecular Mask Strategy (SMS) to Achieve Regioselectivity

Achieving regioselective functionalization of fullerenes remains a significantly greater challenge. Fullerenes are spherical, highly symmetric molecules featuring numerous chemically equivalent double bonds; as a result, functionalization reactions typically yield complex mixtures of polyadducts with poor regioselectivity, which again require difficult and often unsuccessful chromatographic separations. Consequently, controlling where addends attach during fullerene functionalization remains one of the most formidable challenges in fullerene chemistry. Over the past three decades, tether-directed regiofunctionalization^18^—where the length of the tether dictates the regioisomer formed—has proven successful, albeit synthetically demanding and often impractical. To address this issue, we developed a pioneering Supramolecular Mask Strategy (SMS) aimed at the synthesis of isomer-pure polyfunctionalized fullerenes. This approach relies on a host molecule with high affinity for the fullerene guest that selectively shields portions of the fullerene surface, thereby exposing only specific regions for chemical modification ­(Figure 5a). We evaluated the SMS concept using our fullerene-encapsulated assemblies within tetragonal prismatic nanocages, whose geometry features four cross-shaped windows. The exceptional stability and well-defined structures of these host–guest complexes prompted us to explore itero- and regioselective functionalization of encapsulated C_60_ and C_70_. The SMS is reliable, provided the host withstands the experimental conditions used for the fullerene functionalization.

**Figure 5:**
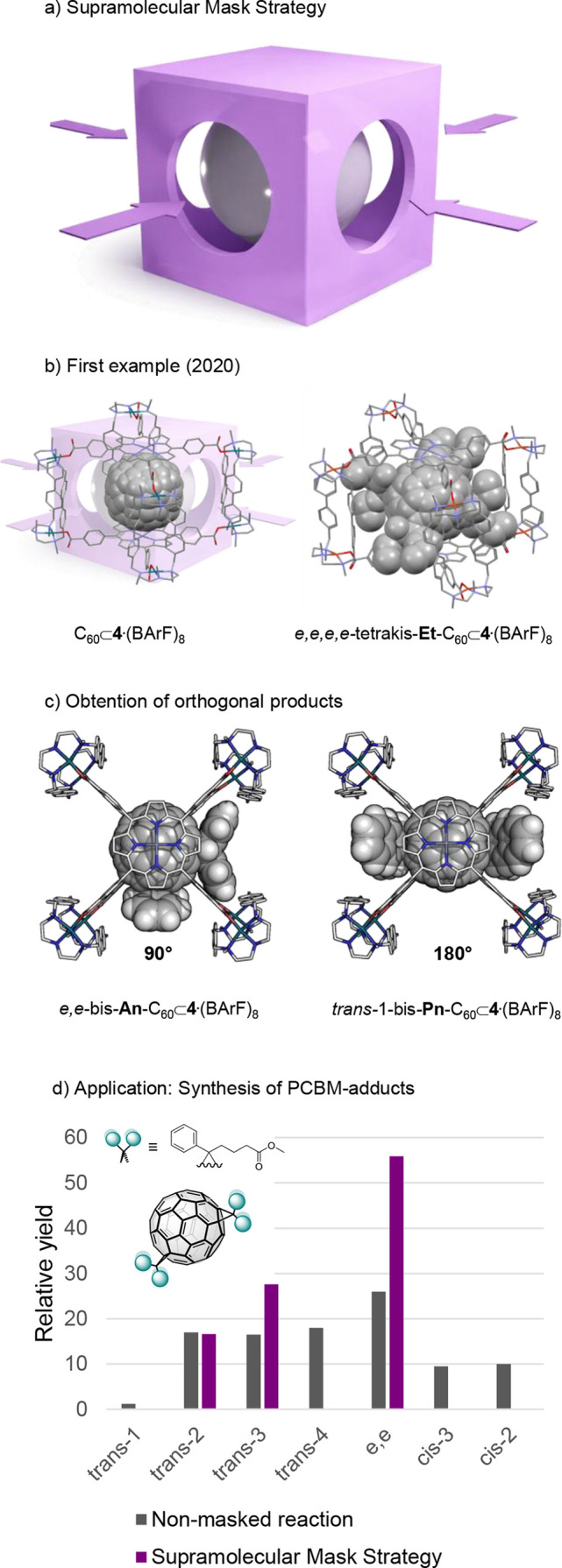
(a) Graphical representation of the SMS. (b) First reported application of the SMS to Bingel regiofunctionalization of C_60_. (c) Orthogonal products obtained from a Diels–Alder reaction on C_60_. (d) 2-fold enhancement in the formation of equatorial bis-PCBM adducts achieved using the SMS.

**Figure 6:**
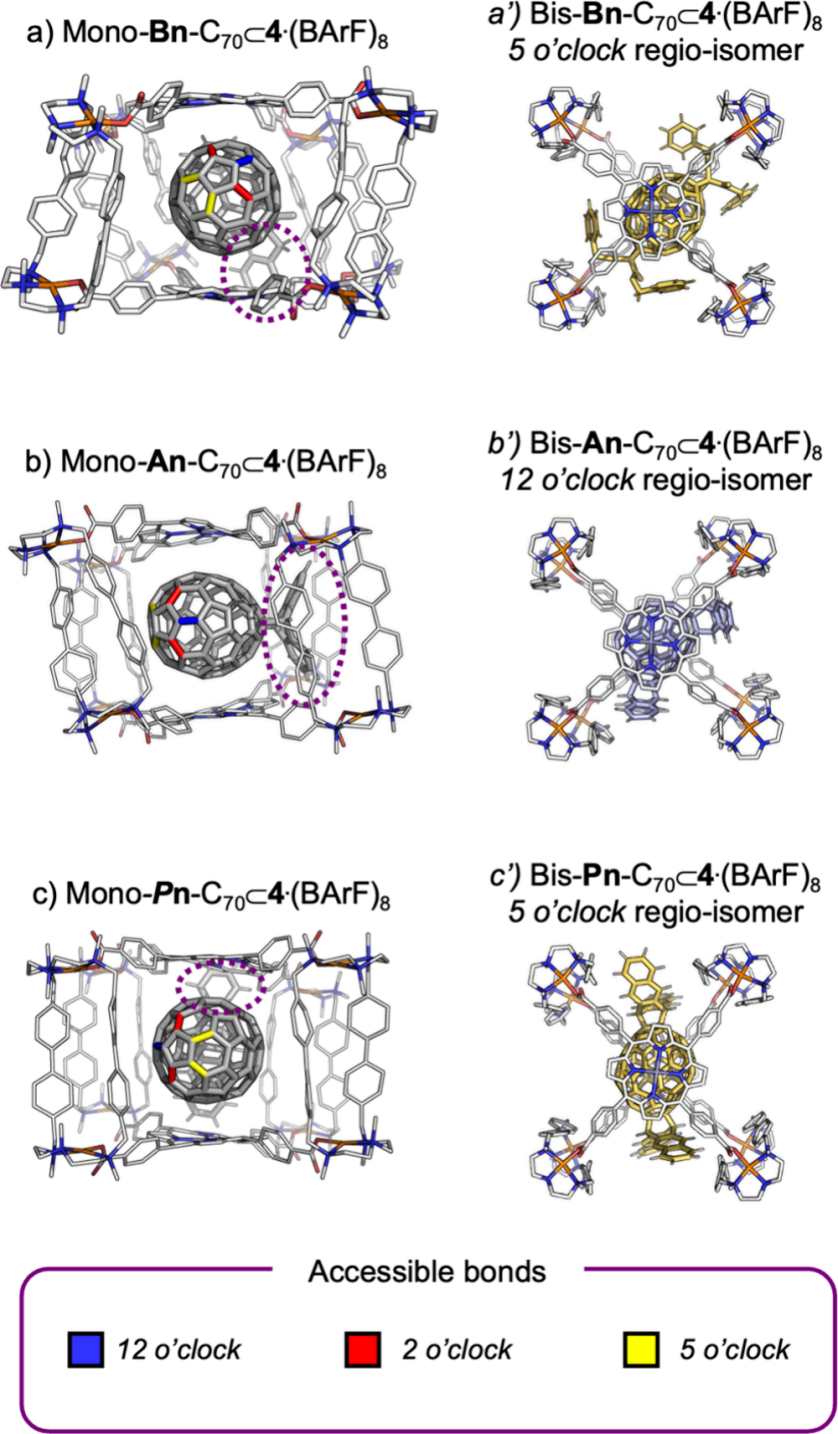
(a–c) Host–guest interactions between the monoadducts and the nanocapsule (highlighted by purple dotted circles), which determine the accessibility of the 12, 5, and 2 o’clock bonds during the Bingel and DA bis-functionalization of C_70_. (a′–c′) Corresponding host–guest complexes of the C_70_ bis-adducts formed inside the nanocapsule.

### 4.1 Supramolecular Mask Strategy Applied to C_60_

Taking advantage of the high stability of the C_60_⊂**4**·(BArF)_8_ host–guest complex, we subjected it to Bingel cyclopropanation conditions. Owing to the octacationic nature of the nanocapsule and its excellent solubility in polar solvents, the reaction was carried out at room temperature in MeCN, using diethyl bromomalonate (4 equiv) and NaH as the base. After 2 h, a single product was detected by HR-MS: the encapsulated tetrakis­(diethyl malonate)–C_60_ adduct (Figure 5b).^14^ The product could be released from the nanocapsule either by competitive exchange with pristine C_60_ or by acid-induced disassembly of the host, which can subsequently be reassembled and reused. Product characterization revealed the exclusive formation of the *D*_*2*_ *h*-symmetric e,e,e,e-regioisomer, obtained in 99% yield and >99.5% purity. Notably, the same reaction performed with free C_60_ does not proceed in MeCN due to the poor solubility of C_60_ in this solvent. When conducted in toluene, the reaction instead affords a complex mixture of bis- to hexakis-adducts within shorter reaction times (30 min), while up to 60% of the starting C_60_ remains unreacted. This control experiment demonstrates that the supramolecular mask profoundly alters not only the regioselectivity, directing nucleophilic attack exclusively toward the equatorial [6,6] bonds, but also the itero- and chemoselectivity, thereby governing both the number of additions and the overall reaction outcome.

The XRD structure of the tetrakis-adduct encapsulated within the Cu-based analogue **5**·(BArF)_8_ revealed that each diethyl malonate group is oriented toward one of the four apertures of the nanocapsule. The relatively short Zn···Zn distance (13.2 Å) highlights the adaptability of the nanocapsule in maximizing porphyrin–fullerene interactions. Molecular dynamics (MD) simulations further showed that rotation of the guest within the cavity is severely restricted, as each diethyl malonate addend is tightly fixed within a single aperture of the nanocapsule. In addition, the malonate addends engage in further noncovalent stabilization through interactions with the nanocapsule clips, establishing persistent C–H···π interactions between the alkyl ester groups and the aromatic rings of the clips. Collectively, the four cross-shaped open windows of the supramolecular mask are crucial for achieving complete control over the functionalization of encapsulated C_60_, governing both regioselectivity (exclusive equatorial addition) and itero-selectivity.

The same SMS can be applied to the synthesis of PCBM-based bis-adducts^35^ using semistabilized sulfur ylides under milder conditions than classical protocols, which typically require diazo compounds and elevated temperatures. Employing the supramolecular mask **4**·(BArF)_8_ increased the proportion of equatorial bis-adducts from 26% to 56% relative to the nontemplated reaction (Figure 5d), demonstrating the broad applicability of this strategy for the preparation of functional PCBM-based materials.

When the functionalization mode is switched from Bingel cyclopropanation to Diels–Alder (DA) cycloaddition, additional parameters—most notably the steric bulk of the acene dienophile—become critical. Performing DA bis-functionalization on C_60_⊂**4**·(BArF)_8_ enables orthogonal regioselective control (Figure 5c), allowing a switch from 90° (*e,e*-bis-**An–**C_60_) using anthracene (**An**) to 180° (*trans*-1-bis-**Pn–**C_60_) using pentacene (**Pn**) simply by increasing the size of the acene.^15^ The origin of this orthogonal regioselectivity lies in the distinct accommodation of the corresponding monoadducts within the supramolecular mask, which differentially restricts access to reactive sites for the second DA addition. MD simulations of mono-**An–**C_60_ encapsulated within the nanocapsule revealed that gate-to-gate rotation of the anthracene addend is possible. Consequently, the kinetically favored second DA reaction occurs at an equatorial position, leading exclusively to the observed *e,e*-bis-**An–**C_60_. In contrast, increasing the steric bulk of the acene imposes greater conformational restriction. MD simulations showed that gate-to-gate rotation of the pentacene moiety in mono-**Pn–**C_60_ is completely suppressed, and the monoadduct adopts a single vertical orientation within the nanocapsule. Because the pentacene unit is forced into a perpendicular arrangement relative to the porphyrin planes, contiguous window access is precluded, and the second DA addition occurs exclusively at the opposite window, yielding the *trans*-1 bis-adduct. Overall, the orthogonal regiofunctionalization of C_60_—producing either *trans*-1 bis-**Pn** or equatorial *e,e*-bis-**An** adducts—is governed solely by the host–guest equilibria of the monoadducts, which are dictated by the complementarity between the acene addend and the supramolecular mask. This highlights the critical role of addend–mask matching in achieving precise regioselective control.

### 4.2 Supramolecular Mask Strategy Applied to C_70_

At this stage, we extended the SMS to C_70_, a guest of increased structural complexity due to its rugby-ball ellipsoidal shape and the second most abundant fullerene obtained from soot extracts. Regioselective bis-functionalization of the less symmetric C_70_ framework presents a significantly greater challenge than that of spherical C_60_. We applied for the first time the SMS to C_70_ using **4**·(BArF)_8_ as the host and Bingel cyclopropanation reaction as the functionalization method. For C_70_, this reaction can yield three constitutionally distinct bis-adducts, commonly referred to as the 2 o’clock, 5 o’clock, and 12 o’clock isomers. Focusing on the dibenzyl bromomalonate (**Bn**-Br) case, the reaction performed on free C_70_ preferentially affords the 2 o’clock isomer (69%), whereas the use of the **4**·(BArF)_8_ supramolecular mask reverses this selectivity, favoring the 5 o’clock isomer with a 77% ratio.^17^ MD simulations of the mono-**Bn–**C_70_ adduct encapsulated within the nanocapsule revealed a markedly reduced rotational freedom compared to unfunctionalized C_70_. The dibenzyl addend is stabilized between two clips, effectively fixing the orientation of the fullerene and exposing the opposite pole through the window across (Figure 6a). Consequently, bis-functionalization is directed preferentially toward the 5 o’clock position, while the alternative sites (2 o’clock and 12 o’clock) are either oriented toward a porphyrin face or shielded by the clips. Once again, these subtle monoadduct–host interactions are crucial in dictating the observed regioselectivity.

**Figure 7:**
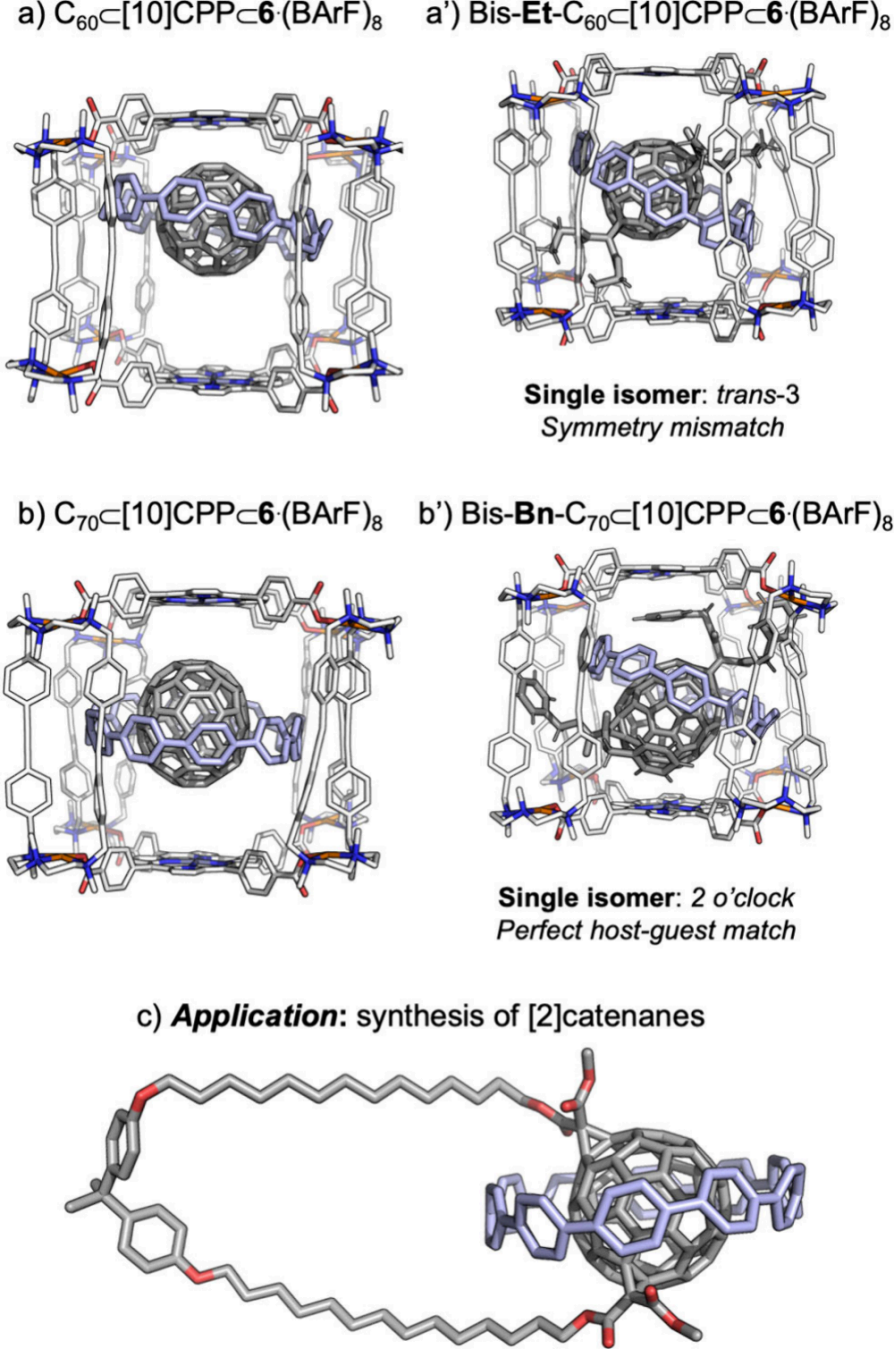
Matryoshka-like complexes with C_60_ (a) and C_70_ (b) and the corresponding bis-adducts obtained upon functionalization (a′, b′). (c) Application of the three-shell assembly in the synthesis of [2]­catenanes.

Extending this strategy to DA cycloaddition of C_70_ with acenes, we observed a regio-divergent outcome that depends on the length of the acene, mirroring the behavior previously observed for C_60_.^36^ Reaction of C_70_⊂**4**·(BArF)_8_ with anthracene (**An**) afforded the 12 o’clock isomer as the major product (>90%). Structural insight from XRD analysis revealed a tight accommodation of the anthracene addends at contiguous windows of the nanocapsule, with the addends positioned at 120° and establishing close contacts with the clip walls of adjacent apertures (Figure 6b). The relatively small size of the anthracene units enables these stabilizing contacts, which contribute favorably to the stability of the host–guest complex. In contrast, when the larger pentacene (**Pn**) was employed, the reaction proceeded with exclusive regioselectivity, yielding 5 o’clock **Pn**_2_–C_70_ as the sole product (>95%). The synergistic interplay between the supramolecular masking effect and the increased steric demand of pentacene results in a matched regioselective outcome, almost completely suppressing formation of the 2 o’clock isomer, which predominates in reactions of unencapsulated C_70_. MD simulations of the monoadducts revealed that the supramolecular mask effectively blocks access to the 2 o’clock position in both the anthracene and pentacene reactions. Overall, shape complementarity between the cage cavity, gate geometry, and acene length directs formation of the thermodynamically favored bis-DA C_70_ regioisomer in each case: the smaller anthracene favors the 12 o’clock isomer, whereas the larger pentacene preferentially yields 5 o’clock **Pn**_2_–C_70_ (Figure 6c). Finally, the nanocapsule could be exploited as a regioisomer converter, taking advantage of the inherent retro-DA equilibrium. Under these conditions, the supramolecular mask quantitatively transformed mixtures of independently synthesized 2 o’clock and 5 o’clock bis-**An**_2_–C_70_ into the 12 o’clock bis-**An**_2_–C_70_ isomer.

### 4.3 Sophisticating the Concept of Supramolecular Mask: Three-Shell Matryoshka-like Complexes

The pivotal role of host–guest interactions between the monoadduct and the nanocapsule in directing regioselectivity prompted us to explore a more complex system, namely a three-shell masking self-assembly. Such architectures—often referred to as molecular Russian dolls or Matryoshkas—have proven valuable for probing the limits of supramolecular chemistry, although practical applications of these sophisticated constructs remain scarce. Our approach involved combining our supramolecular nanocapsules with an additional carbon cycloparaphenylene (CPP) nanoring surrounding C_60_. Functionalization of C_60_ in the presence of a [10]­CPP nanohoop alone has been shown to impose some degree of regioselective control, as reported by the von Delius group, favoring mainly the *trans*-2 and *trans*-3 isomers.^37^

Accordingly, we set out to construct a three-shell Matryoshka assembly (C_60_⊂[10]­CPP⊂cage) to investigate the outcome of Bingel cyclopropanation under conditions of severely restricted access to the fullerene surface. Accommodating the C_60_⊂[10]­CPP complex required a nanocapsule with an enlarged cavity; therefore, the **6**·(BArF)_8_ cage was designed and synthesized (Figure 2). The crystal structure of C_60_⊂[10]­CPP⊂**6**·(BArF)_8_ revealed two equally populated guest orientations (50% occupancy), with interactions between the Zn porphyrins and the fullerene being prioritized. In the solid state, the [10]­CPP nanohoop adopts a slightly tilted orientation (≈15°) relative to the porphyrin planes. As a result, many potential angles of reagent approach are blocked: the bromomalonate reagent must enter through one of the four nanocapsule windows and, once inside, avoid steric clashes with the [10]­CPP ring. Under these constraints, the Bingel reaction afforded a three-shell complex containing a C_60_ bis-adduct in an itero-selective manner. Release of the bis-adduct was achieved by displacement from the [10]­CPP ring through the addition of excess pristine C_60_, thereby regenerating a reusable Matryoshka assembly. Product analysis unequivocally identified a single bis-adduct regioisomer, namely *trans* −3-C_60_.^16^ At first glance, this regioisomer is counterintuitive: the *trans* −3-C_60_ bis-adduct features a 120° angle between the two addends, whereas the nanocapsule exhibits 4-fold symmetry, corresponding to 90° separation between the windows. However, in this multicomponent system, there is only one geometrically viable arrangement that accommodates all components simultaneously, which involves reagent access through contiguous windows separated by 120° (Figure 7a). By combining two distinct supramolecular masking strategies, we achieved an unpredictable regioisomeric outcome, representing a rare and striking example of symmetry mismatch between a supramolecular nanocontainer and the resulting reaction product.

When the same strategy was applied to C_70_⊂[10]­CPP⊂**6**·(BArF)_8_, excellent control over bis-Bingel functionalization was also achieved,^17^ with the 2 o’clock regioisomer emerging as the favored product—contrasting with C_70_⊂**4**·(BArF)_8_, which preferentially yields the 5 o’clock isomer. In the specific case of dibenzyl bromomalonate (**Bn**-Br), an optimal match between the nanocapsule architecture, the orientation of the [10]­CPP ring, and the nature of the addend resulted in complete regioselectivity (100%) for the 2 o’clock isomer (Figure 7b). Notably, one of the benzyl addends in the product is sandwiched between the porphyrin and the fullerene surface, providing additional stabilization that favors this regioisomer. These results demonstrate that regioselectivity can be finely tuned by modifying either the supramolecular mask or the addend structure.

In this case, the host does not simply transfer symmetry to the guest; rather, the hierarchical assembly creates a confined environment that selectively stabilizes a specific addition pattern not intrinsically favored by the nanocapsule, the [10]­CPP, or the guest itself. More broadly, such a symmetry mismatch between host and product may represent a general strategy for accessing unconventional regioisomeric outcomes in highly symmetric substrates. By combining complementary, or competing, supramolecular constraints within hierarchical architectures, unexpected yet high regioselective outcomes may arise. In this context, observed addition patterns cannot be predicted from individual host–guest interactions, but emerge from the cooperative interplay among all components of the multilayered supramolecular architecture.

The Matryoshka-like complex C_60_⊂[10]­CPP⊂**6**·(BArF)_8_, was also applied to the synthesis of [2]­catenanes incorporating both C_60_ and the [10]­CPP ring (Figure 7c).^38^ In this approach, combining the well-established tether strategy^18^ -starting from a precursor in which two malonate units are covalently linked- with our SMS enabled access to a mechanically interlocked architecture that would otherwise be inaccessible.

## 5 Summary and Prospectus

This Account summarizes our development of supramolecular nanocapsules capable of controlling both fullerene recognition and reactivity via confinement within a well-defined host architecture. These systems enable selective encapsulation of fullerenes (C_60_, C_70_) and higher fullerenes (C_84_, fullertubes) and, through our pioneering Supramolecular Mask Strategy (SMS), can provide access to isomerically pure polyfunctionalized fullerenes. Detailed studies of the binding modes between the host and pristine or functionalized fullerene rationalize the experimental outcomes and highlight the capabilities of nanocapsules as confined reaction environments.

From this work, several general design principles for supramolecular nanocapsule control of fullerene selectivity and reactivity emerge:

(i) precise matching between the cavity dimensions (e.g., Zn···Zn distance) and guest size governs encapsulation selectivity and, by defining the accessible surface through window size and geometry, also directs the regioselectivity of subsequent functionalization.
(ii) Host–guest interactions are crucial for encapsulation selectivity; nanocapsule design should incorporate recognition points for the fullerene guest (e.g., porphyrin units). Postbinding interactions can further stabilize the encapsulated guest (e.g., clamping motion in the case of fullertubes). Moreover, interactions between the host and monoadduct can steer iterative reactions and subsequent regioselective additions.
(iii) Multilayer confinement opens new opportunities for selective encapsulation and regioselective functionalization of fullerenes. Large cavities accommodating preformed host–guest complexes can form hierarchical assemblies that generate unique reaction environments, differing from those of the individual components. As a result, regioselective outcomes may emerge that are not predictable from each supramolecular layer taken separately. Instead, the overall architecture and the cooperative interplay among all layers define the reaction landscape, enabling highly selective and unconventional functionalization patterns.

The dynamic adaptability of these nanocapsules highlights the advantages of encapsulating guests of different sizes, while also revealing the limitations of lacking exclusive size-selectivity. Leveraging our understanding of the inner cavities of this family of nanocapsules and their binding modes with spheroidal, ellipsoidal, and tubular fullerenes, we envision that strategic functionalization of the cavity could further enhance selectivity for specific fullerene guests and enable discrimination among competing substrates and isomers.

The demonstrated applicability of these nanocapsules for encapsulation and controlled functionalization of fullerenes of varying sizes and shapes suggests that analogous strategies may be extended to nonfullerene guests, provided an effective anchoring motif is available. Potential targets include nanotubes, metal clusters, polycyclic aromatic hydrocarbons (PAH), spherical molecules, and biologically relevant macromolecules. A promising future direction involves the design of multilayer supramolecular systems that create increasingly complex confined environments, unlocking previously inaccessible reactivity. By integrating multiple, hierarchically organized recognition and confinement elements, such architectures could enable new levels of selectivity control.

A deeper understanding of confined space interactions and recognition pathways will be essential for developing supramolecular containers with predictable binding affinities and programmable reactivity. Integrating selective recognition with confinement-controlled transformations offers a promising route toward predictive and programmable nanoscale chemistry. Supramolecular encapsulation introduces a new dimension of selectivity, rooted in a careful analysis of the host architecture.
